# Effect of axial misalignment and tip clearance on the performance of double spiral seals

**DOI:** 10.1371/journal.pone.0314912

**Published:** 2024-12-05

**Authors:** Haorui Liao, Ning Li, Feng Gao, Yuzhou Ming, Xingyun Jia, Kening Li, Decai Bian

**Affiliations:** 1 Beijing University of Chemical Technology, College of Mechanical and Electrical Engineering, Beijing, China; 2 Science and Technology on Helicopter Transmission Laboratory, Zhuzhou, Hunan, China; 3 AECC Hunan Aviation Powerplant Research Institute, Zhuzhou, Hunan, China; 4 China Institute of Atomic Energy, Beijing, China; GH Raisoni College of Engineering and Management Pune, INDIA

## Abstract

The axial misalignment and tip clearance of the double spiral seals are important indexes affecting the sealing performance of the double spiral seals. In practical engineering applications, the sealing ability of double spiral seals is greatly affected by axial misalignment and tip clearance, and the coupling of assembly parameters and structural parameters of double spiral seals leads to the difficulty of seal design and use. A numerical model of spiral seals was established, and the velocity field, pressure field and critical sealing ability of double spiral seals were simulated and calculated. The influence of different axial misalignment displacements and different tip clearances on the sealing ability of double spiral seals was analyzed. The influence of axial misalignment displacement, tip clearance and rotor speed on the sealing performance of double spiral seals is obtained. The above research results provide a design basis for the engineering application of double spiral seals.

## 1. Introduction

Spiral seals are divided into single spiral seals and double spiral seals, both of which are non-contact dynamic seals. The principle of the spiral seal is that when the shaft rotates, the spiral groove will produce an axial force with an inverse pressure difference. Under the action of the axial force, the medium forms a reverse flow to balance the pressure difference at both ends of the seal, so that the working medium does not leak. The double-helix seal has a simple structure, and there is a gap between the dynamic and static rings, so that the stator and the rotor will not rub. It can produce a large sealing force at high speed [1], so as to achieve zero leakage. It is very suitable for harsh conditions such as high temperature, deep cold, corrosion and medium with particles, and has a good application prospect. The application scope of double spiral seal in industry mainly includes double spiral feeder, spiral pump, and under the conditions of high temperature, high pressure and corrosive medium. Since the double-helix seal is often used in practice, the sealing performance is changed and even leakage occurs due to the axial misalignment and the difference in the tip clearance. Therefore, it is necessary to calculate and analyze the influence of different axial dislocation displacements and tip clearances on the sealing performance of double-helix seals, so as to provide theoretical reference for the design, installation and use of double-helix seals, so as to accurately improve the sealing ability of double-helix seals.

At present, the research on the spiral seal is mainly focused on the study of the mechanism and the study of the structural parameters. Tong Li et al. [2] used finite volume software to construct a numerical model of spiral seal, and analyzed the pressure change and velocity distribution of its internal flow field in detail, thus revealing the sealing mechanism of spiral seal. By constructing a numerical model of spiral seals, Zheng Rao et al. [3] studied the influence of different structural parameters on the performance of single spiral seals. These structural parameters include tip clearance, thread tooth height and relative groove width. The research shows that with the increase of the tip clearance, the sealing performance of the spiral seal will gradually deteriorate from good to bad until leakage occurs. Feng Ruipeng [4] deeply studied the influence of the structural parameters of the double-helix dynamic pressure seal end face on the sealing performance under the condition of high operating speed. These parameters include spiral angle, groove depth, groove number and inner-outer groove diameter ratio. Based on the research foundation of Che ’s spiral seal, Liu Jie [5] studied the specific influence of medium spiral length, tooth width and tooth height parameters on leakage under high pressure conditions. Ma Runmei [6] regarded the spiral seal as an axial flow pump with a long axial tooth, and reasonably explained the working mechanism of the spiral seal and the phenomenon of ’ air swallow ’ and ’ seal failure ’. Gaoyuan et al. [7] studied the performance of labyrinth-like spiral seal and labyrinth seal by finite volume software, and found that the sealing performance of the two seals was basically the same, which proved the feasibility of using labyrinth-like spiral seal. Bo Xiangfeng et al. [8] used a self-designed and processed test device to compare different spiral structure parameters such as spiral angle and spiral groove width of spiral seal, and concluded that the sealing ability of spiral seal will be greatly reduced under a certain set of parameters. Wang Rui et al. [9] simulated and analyzed the pumping flow and leakage flow through finite volume software, and obtained the law of the influence of different process parameters on the sealing ability of spiral seal. Liu Zhong et al. [10] used the finite volume method to optimize the structural parameters of the double spiral groove end face seal, such as the groove depth and the number of grooves. For a new type of double spiral groove end face seal device, Liu Ke et al. [11] studied the influence of spiral angle on sealing performance. Paudel, Wisher et al. [12] analyzed the mechanism of labyrinth-spiral hybrid seal, simulated the combined seal design of labyrinth rotor and spiral stator, and studied whether the optimal number of grooves, groove width and groove depth changed due to the spiral stator. The research shows that the mixed spiral labyrinth seal is effective under both low pressure and high pressure. Zhang Juqian [13] found that for a single spiral seal, under the action of differential pressure force, there are axial leakage along the annular clearance of the tooth tip clearance and leakage along the spiral groove. When the ratio of the tooth tip width to the tooth groove width of the spiral seal structure is 1, the tooth groove depth and the clearance ratio are 2.61, the sealing ability is the strongest. Li Yibin et al. [14] established a coupled three-dimensional model and block structured mesh model including centrifugal pump and spiral seal, and found the influence of working parameters such as spiral angle, relative groove width and relative groove depth on the sealing performance of spiral seal. Ai Renjie [15] numerically simulated the leakage of spiral seals under different structural parameters and different operating parameters, and found the influence of spiral angle, relative groove depth and rotor speed on the leakage characteristics of spiral seals. Zhou Zexin [16] studied the influence of spiral seal structure parameters such as spiral groove depth, relative groove width, spiral lift angle and seal clearance on the flow field characteristics such as pressure distribution and velocity distribution of turbine pump spiral seal, and then found the influence of spiral seal structure parameters on the leakage characteristics of spiral seal. With the help of Fluent software, Li Jianzhong [17] carried out steady numerical simulation on the internal flow of spiral seal under different parameters, and then found that there were optimal values of spiral angle, relative groove width and relative groove depth under different axial Reynolds numbers. Yang Bing [18] used finite volume software to numerically simulate the spiral seal, and found the variation law of the sealing capacity of the spiral seal when the rotation speed, dynamic viscosity, sealing length, clearance, groove depth, relative groove width and thread angle change.

Previous studies on spiral seal mainly focused on the influence of structural parameters of single spiral seal on sealing performance. These structural parameters include tip clearance, thread tooth height, relative groove width, spiral angle, spiral length, spiral groove width and so on. However, there are few studies on the influence of the main parameters of the double helical seal, such as axial displacement, tip clearance and rotor speed on the sealing performance. Based on the previous studies, this paper starts from the sealing performance of the double-helix seal, and uses FLUENT software to qualitatively and quantitatively analyze the flow characteristics of the fluid in the flow field of the double-helix seal. The variation law of the sealing performance of the double-helix seal under the axial misalignment displacement and the change of the tip clearance is revealed.

## 2. Establish the geometric model of double spiral seals

**Fig 1:**
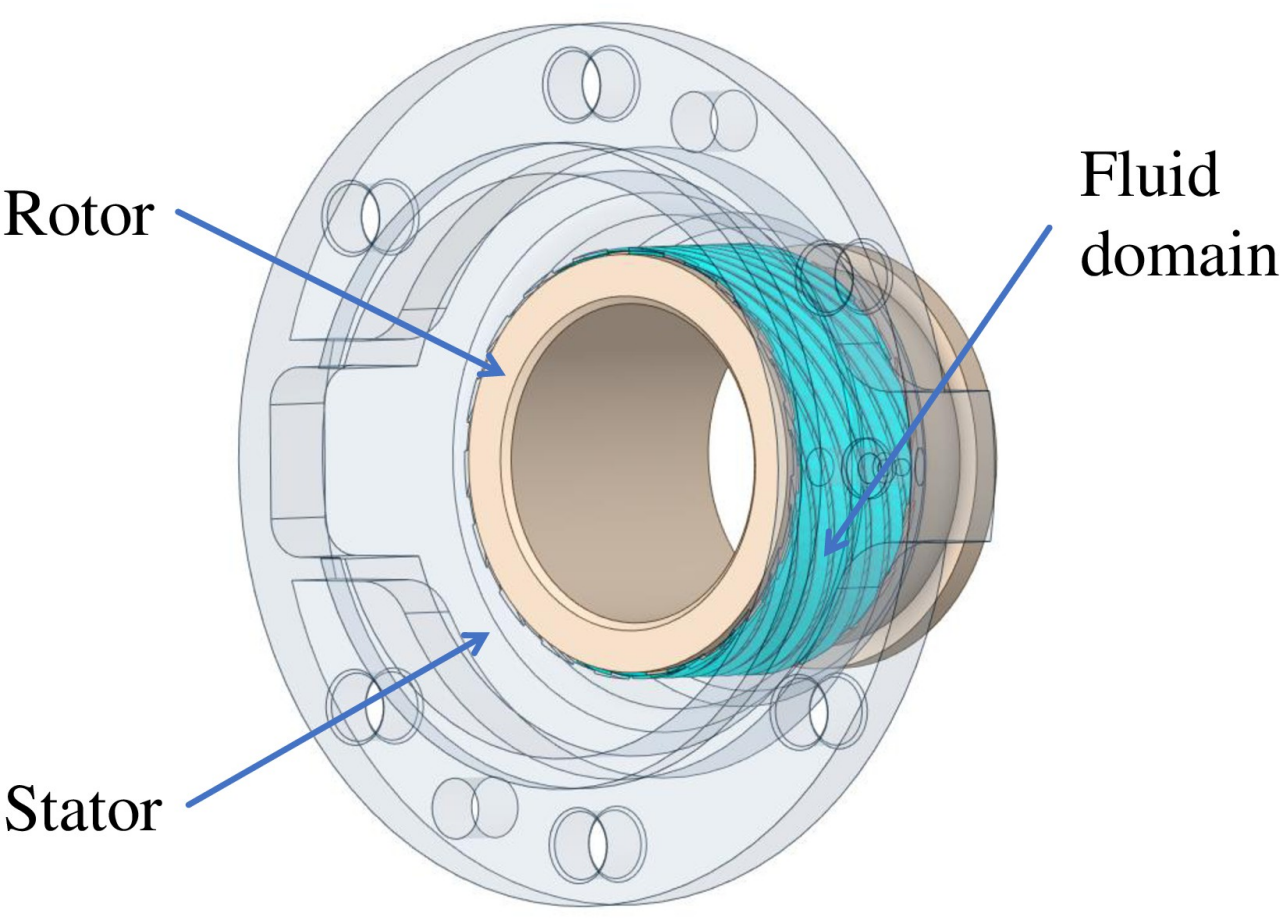
Three-dimensional model of double spiral seal.

**Fig 2:**
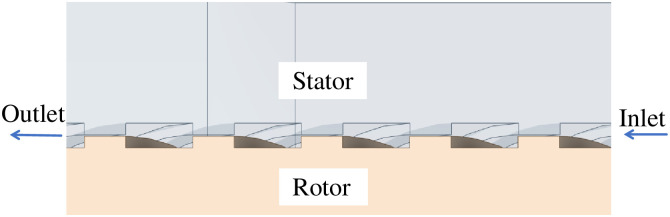
Double spiral seal flow field inlet and outlet diagram.

**Fig 3:**
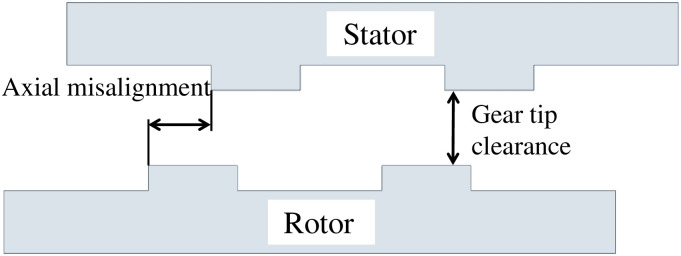
Schematic diagram of double spiral sealing structure.

Due to the complex and changeable medium flow in the double-helix sealing basin, it is necessary to establish an accurate three-dimensional model and select the correct mathematical model for simulation calculation. In this paper, the model of double helix seal is established, and the model is shown in Fig 1, and then the fluid simulation calculation is carried out in finite volume software. The inlet and outlet of the flow field of the double-helix seal are shown in Fig 2. The axial misalignment and tip clearance of the double-helix seal calculated in this paper are shown in Fig 3.

In this paper, the code of axial displacement is s, and the code of gear tip clearance is d. The structural parameters of the double-helix seal model used in this paper are shown in Table 1.

**Table 1:** Structural parameters of double spiral seal.

Name	Numerical value	Name	Numerical value
Thread maximum diameter/mm	55.8	Thread tooth height/mm	0.4
Thread tooth width/mm	1.43	Working length/mm	22.3
Thread groove width/mm	2.3	Spiral angle/°	20.9
Gear tip clearance/mm	0.05	Thread number	18
Thread form	rectangle	Thread orientation	levorotation

## 3. Establish the calculation model of double spiral seal

**Fig 4:**
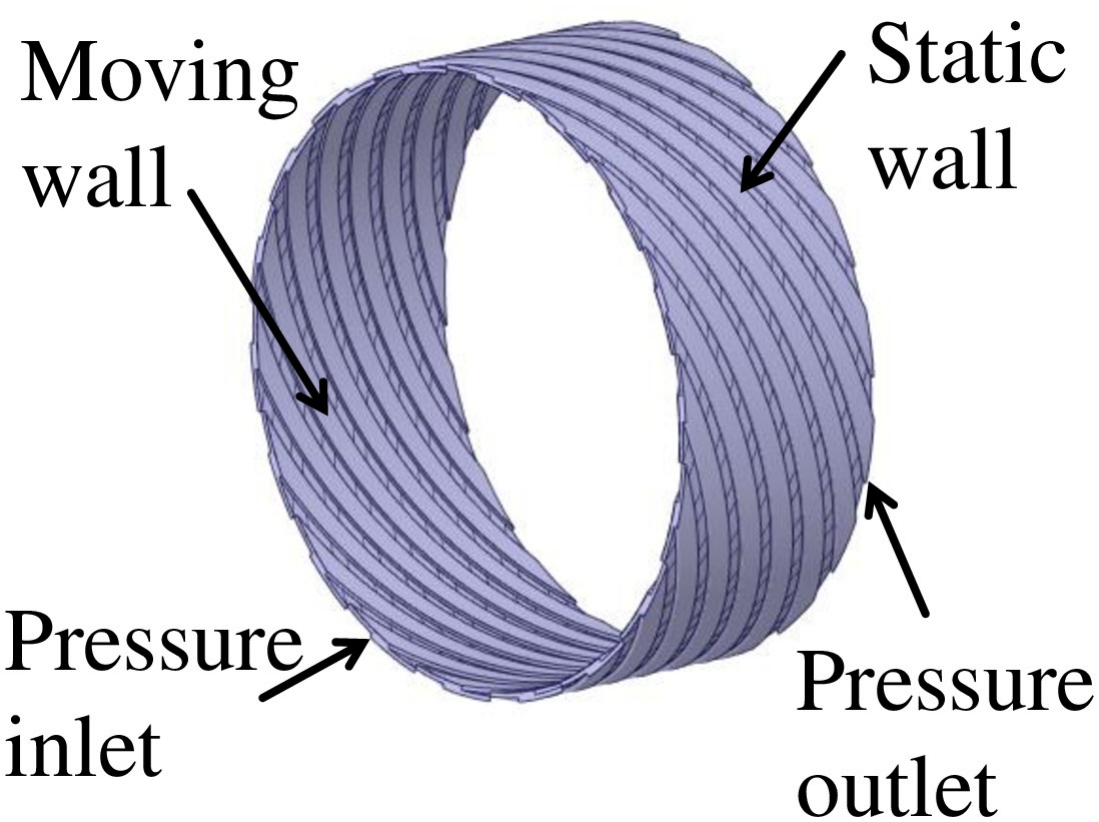
Set the boundary conditions of fluid domain.

**Fig 5:**
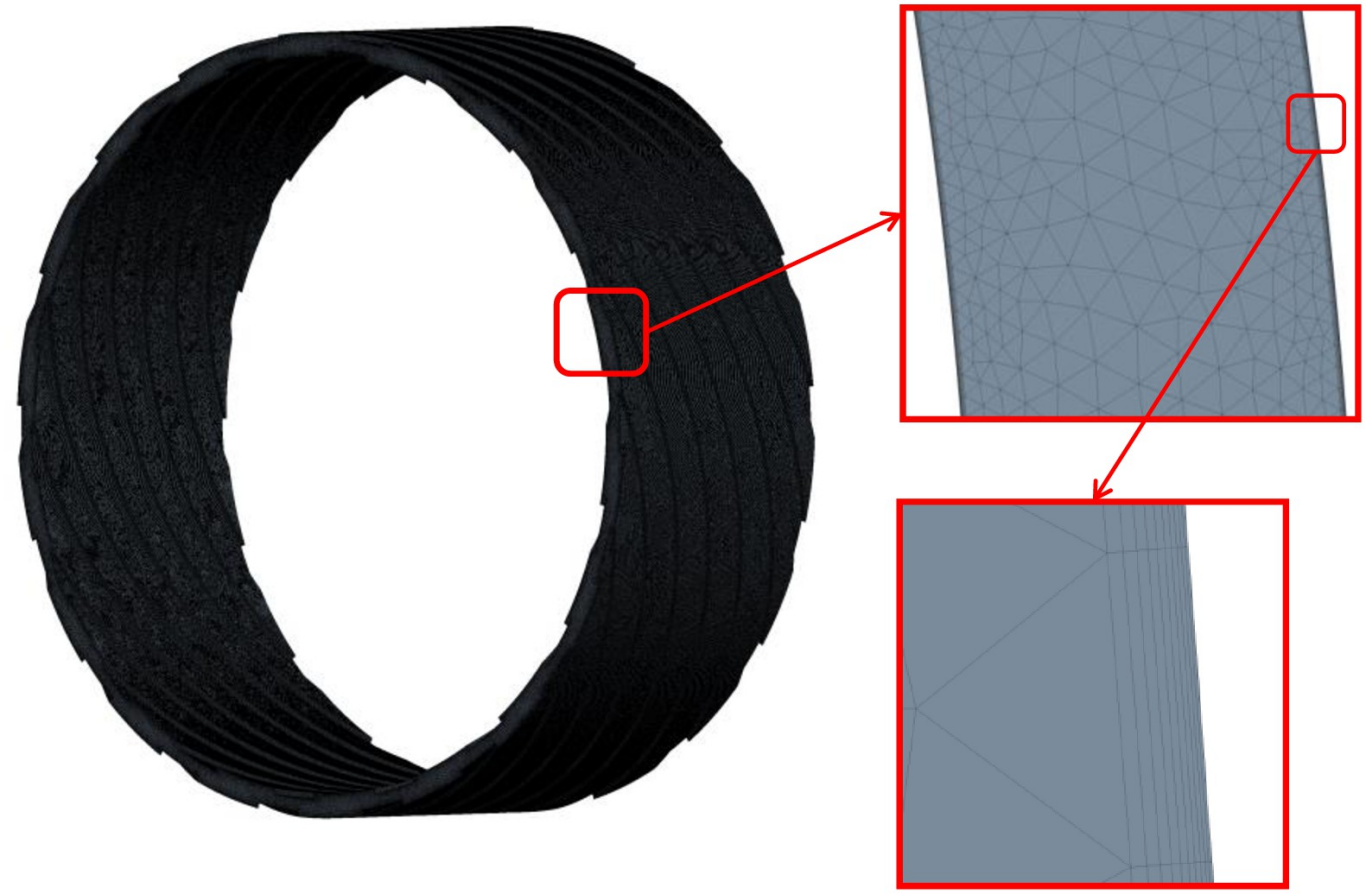
Mesh subdivision.

**Fig 6:**
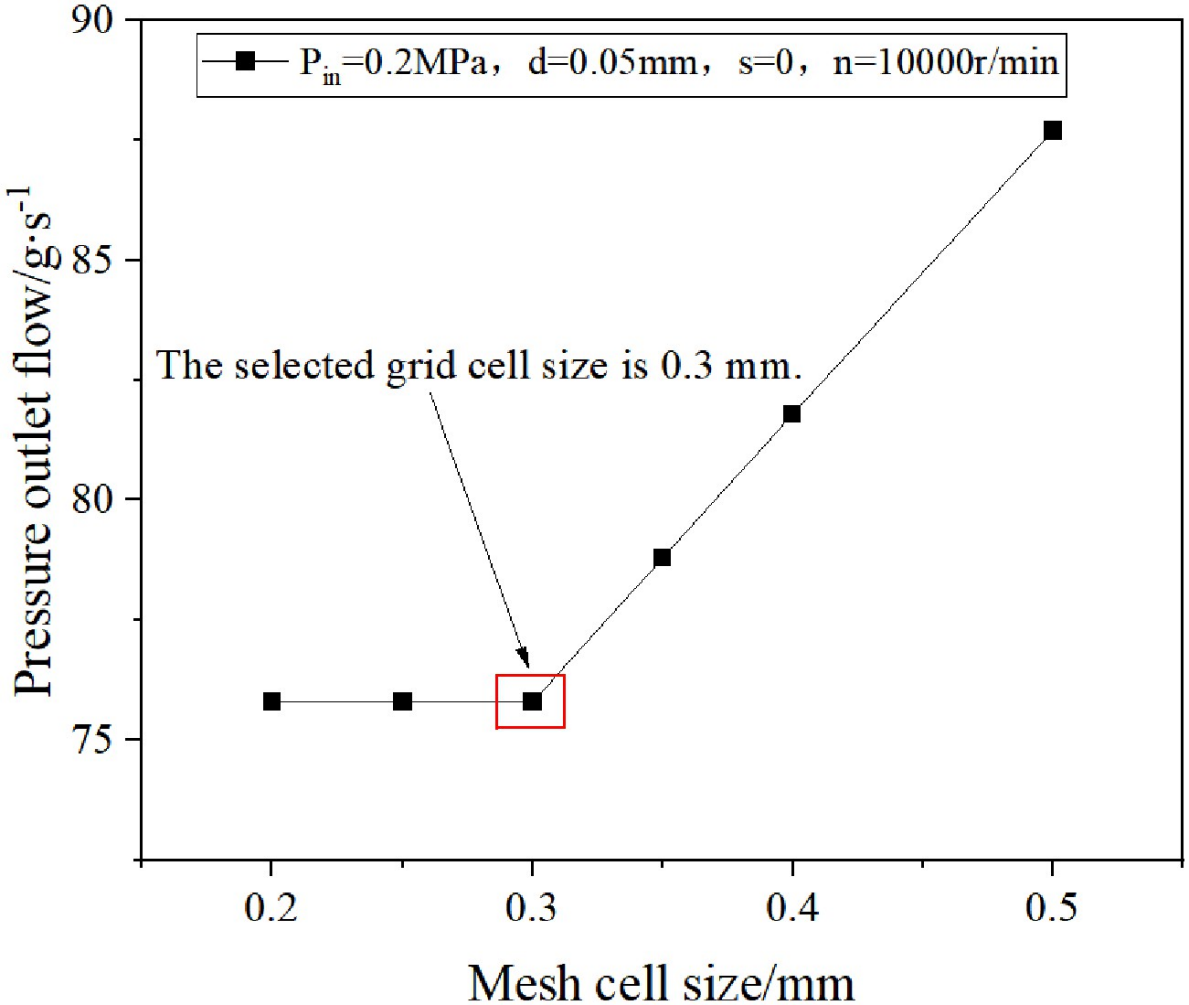
Mesh independence verification.

The research in this paper focuses on the fluid domain between the rotor and the stator in the double-helix seal model, and the corresponding fluid domain analysis model is constructed (as shown in Fig 4). In the setting of boundary conditions, the inlet pressure of the fluid is set to P_in_ = 0~0.45MPa (gauge pressure), and the outlet pressure is set to P_out_ = 0MPa (gauge pressure). In addition, the fluid domain in contact with the rotor is defined as the rotation domain, and its rotation speed is set to be n = 10000r/min, while the fluid domain in contact with the stator remains stationary. At the same time, the mesh module in finite volume software is used to divide the mesh (as shown in Fig 5). After the mesh-independent verification (as shown in Fig 6), the mesh meets the calculation accuracy requirements. In this paper, the mesh geometry is tetrahedral. Tetrahedral mesh is a classical type of unstructured mesh. Tetrahedral mesh is particularly suitable for surface mesh generation of complex geometries. It can automatically generate meshes and generate faster. In addition, tetrahedral meshes can generate a large number of mesh elements when dealing with complex geometric shapes, so as to better adapt to the complexity of geometry. For the double spiral seal model studied in this paper, there are complex structures such as tooth groove and spiral line on the surface. In this paper, the mesh is a tetrahedral mesh and a wall layer is set at the same time, and the mesh refinement is set near the wall area, which can reflect the characteristics of the flow. For the double spiral seal model studied in this paper, there are complex structures such as tooth groove and spiral line on the surface. Due to the particularity of the computational domain structure of the spiral seal fluid, the hexahedral mesh is difficult to divide, and the mesh has an acute angle, which is not well handled, resulting in calculation failure. Therefore, the tetrahedral mesh is used for calculation. After the volume mesh is generated, the boundary layer is set on the inner wall and outer wall. The number of layers of the boundary layer is 10. The height of the first layer is 0.0001 mm according to the formula, and the boundary layer mesh is triangular prism unit. The content of mesh independence test has been shown in Fig 6, the selected mesh cell size is 0.3 mm. The mesh growth rate is 1.2, the maximum number of layers is 10, and the transition ratio is 0.272. Most of the mesh skewness is around 0.4, and the maximum mesh skewness is about 0.6. The number of mesh nodes is 195387 and the number of mesh cells is 779558. The mesh is divided according to the extracted fluid domain, and the mesh diagram is shown in Fig 5. After the mesh is divided, the mesh independence test was carried out. Firstly, starting from the mesh unit size of 0.5mm, the pressure outlet flow under the conditions of mesh unit size of 0.5mm, 0.4mm, 0.35mm, 0.3mm, 0.25mm and 0.2mm is calculated in turn, and then the chart is drawn according to the calculation results, as shown in Fig 6. From Fig 6, it can be seen that as the mesh cell size becomes smaller and smaller, the calculation results gradually become stable. When the mesh cell size is 0.3mm, the pressure outlet flow begins to remain stable. Therefore, the mesh cell size selected in this paper is 0.3mm.

In order to verify the correctness of the simulation analysis using finite volume software, through consulting the literature, it is known that the tip clearance is also one of the structural parameters that affect the sealing performance of the spiral seal. According to the model size and operating parameters provided by the reference, the pressure outlet flow of the single spiral seal under different tip clearances is simulated.

The inlet pressure is set to 0.2MPa, 0.3MPa, 0.45MPa three groups, the inner wall of the fluid is set to the rotating wall, N = 10000 r/min, the tip clearance is 0.05mm, 0.075mm, 0.1mm, 0.125mm, 0.15mm five groups.

**Fig 7:**
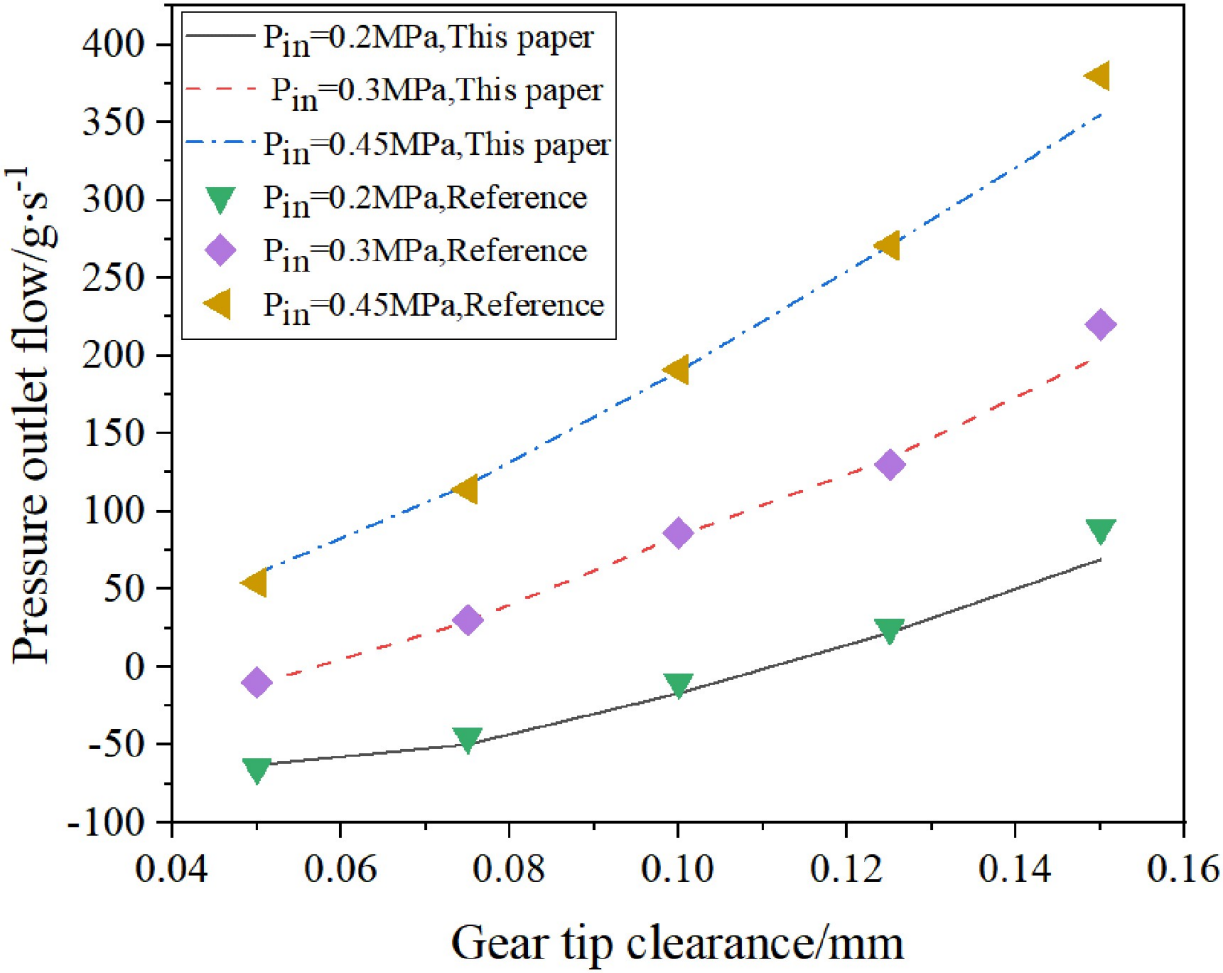
The calculation results are compared with the literature results [3].

According to the calculation results, the influence of the tip clearance on the sealing ability of the single screw seal is drawn and compared with the results of the reference (as shown in Fig 7). Here, when the pressure outlet flow is negative, the sealing ability is good, otherwise, the sealing ability is poor.

It can be seen from Fig 7 that the calculation results are basically consistent with the literature results, and the data error is within a reasonable range, which proves the correctness of the numerical method. The operating parameters used in the simulation of the double-helix sealing model in the finite volume software are shown in Table 2. The turbulence model of Standard k-ε is used for simulation.

**Table 2:** Operating parameters of finite volume software.

Name	Numerical value	Name	Numerical value
Inlet pressure/MPa	0~0.45	medium temperature/K	293
Outlet pressure/MPa	0	Density/kg∙m^-3^	889
Fluid medium	oil	Viscosity/Pa∙s	10.4
Revolution speed/r∙min^-1^	0~10000		

## 4. Flow field calculation and analysis of double spiral seals

### 4.1. The influence of pumping effect on the sealing flow field

In order to better understand the flow law of the double-helix seal flow field, the flow characteristics of the flow field without pumping effect are first studied.

**Fig 8:**
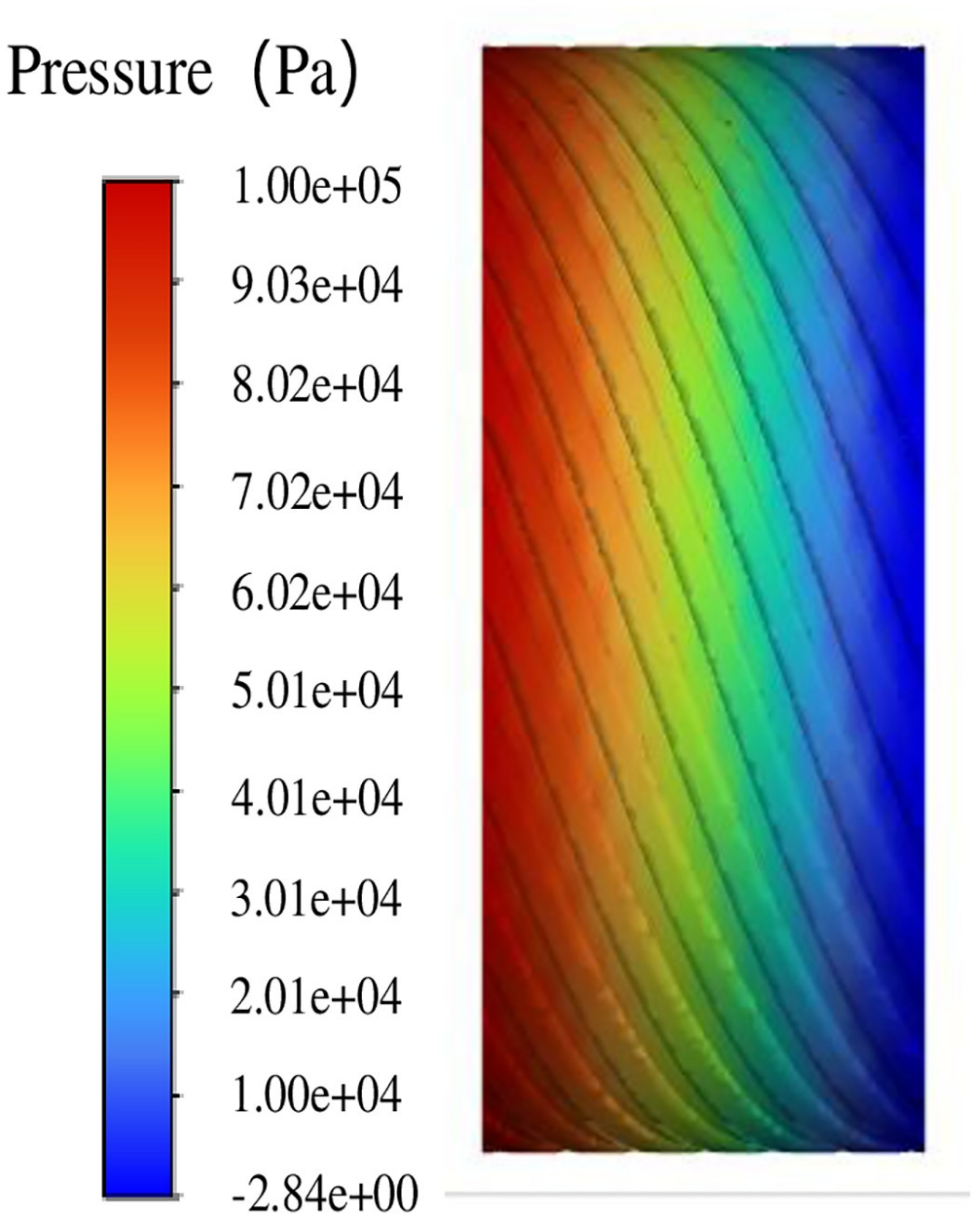
Overall pressure distribution diagram.

The inlet pressure is set to 0.1 MPa (gauge pressure), the outlet pressure is set to 0 (gauge pressure), and the fluid rotation domain is set to 0. In the simulation of leakage flow, because the rotor does not rotate and does not produce pumping flow, only the flow under the action of pressure difference is considered, that is, leakage flow. From the pressure cloud diagram shown in Fig 8, it can be seen that the pressure distribution in the fluid domain of the double helix seal presents significant characteristics under the influence of pressure difference. Specifically, the pressure value of the inlet spiral groove is relatively high, while the pressure of the outlet spiral groove is gradually reduced, and even negative.

**Fig 9:**
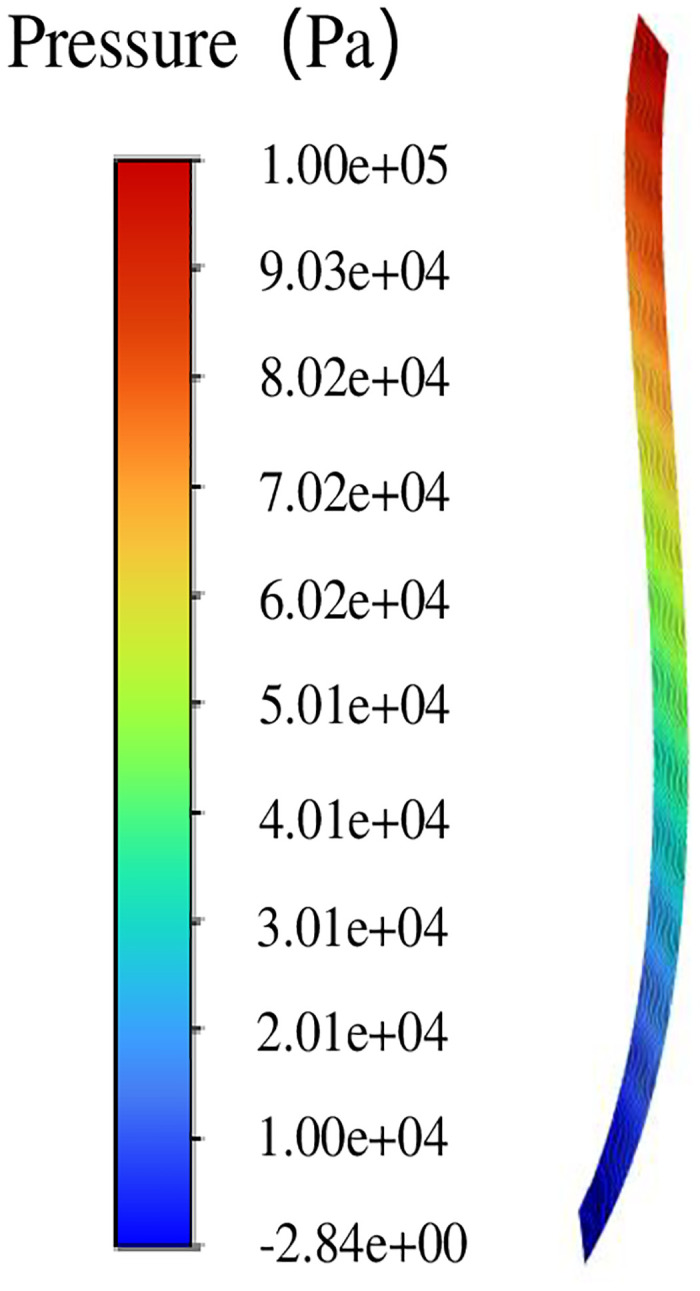
Pressure distribution along the spiral groove.

Fig 9 further reveals the details of pressure changes in the spiral groove. From the inlet to the outlet, the pressure gradually decreases, especially in the spiral groove. Compared with the gap pressure drop between the spiral rotor and the stator, the pressure change in the spiral groove is more significant.

**Fig 10:**
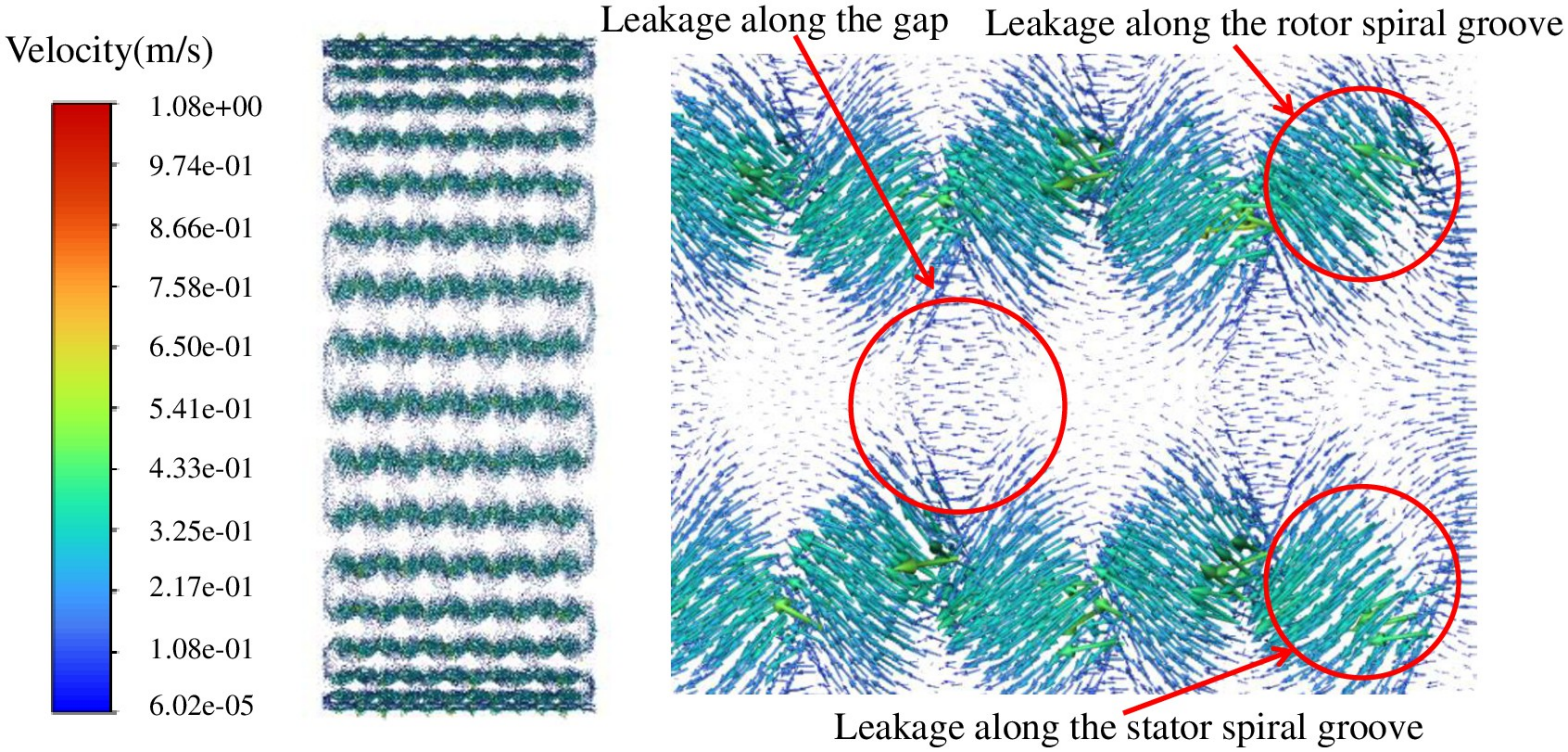
Spiral groove velocity vector diagram.

The spiral groove velocity vector diagram in Fig 10 clearly shows that under the action of pressure difference, the leakage flow does not only occur inside the spiral groove of the rotor and stator, but also occurs in the gap part of the double spiral seal. The leakage flow is divided into two parts: one part occurs inside the spiral groove, and the other part occurs in the gap between the rotor and the stator.

Figs 8–10 show the study of fluid flow characteristics without pumping effect, and the flow law of fluid in the flow field of double spiral seal is basically obtained. Next, the influence of pumping effect on the seal flow field is studied.

**Fig 11:**
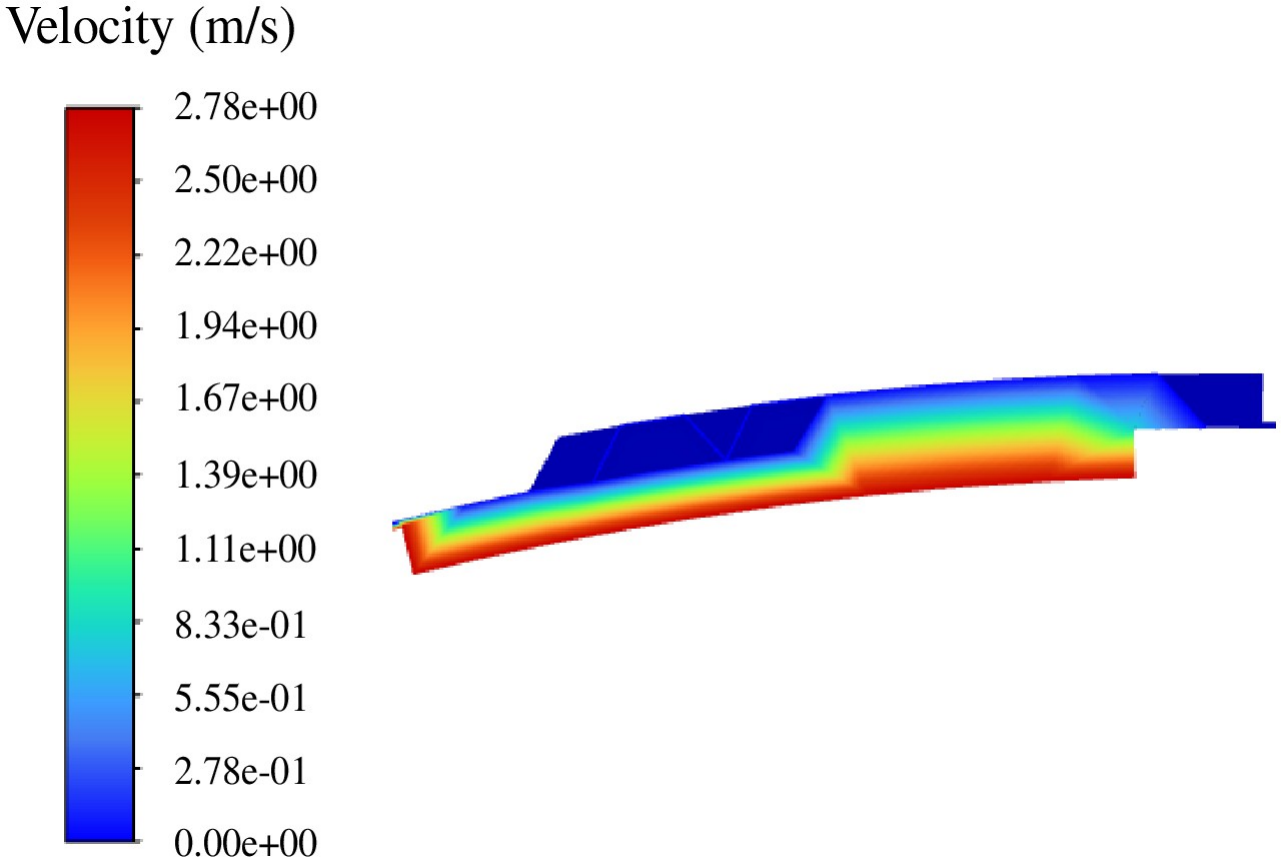
Pumping flow velocity distribution diagram.

The inlet pressure is set to zero, the inner wall of the fluid is set to the rotating wall N = 10000 r/min, and the velocity cloud diagram of the pumping is shown in Fig 11.

It can be seen from the figure that the hydrodynamic force of the double-helix seal mainly depends on the rotation of the rotating shaft under the condition that the pressure difference is 0. Specifically, the flow velocity of the fluid near the spiral axis is larger, and the closer to the outer wall (stator), the flow velocity is gradually reduced, even almost 0.

The reason for this phenomenon is that the rotational motion of the spiral rotor promotes the laminar flow of the fluid in the spiral groove and the fluid in the sealing gap. However, due to the viscous force of the sealing medium itself, the flow velocity gradually decreases as the fluid moves outward from the groove depth.

### 4.2 The influence of axial misalignment on the sealing performance of double helical seals

In fluid dynamics, the relationship between pumping flow and leakage flow is crucial for the sealing performance of double helix seals. When the pumping flow exceeds the leakage flow, it indicates that more fluid is pumped into the system. At this time, the inlet mass flow is negative, while the outlet mass flow is positive. In this case, the double helix seal can provide sufficient sealing capacity at a given differential pressure to ensure that the fluid does not leak from the seal.

However, when the pumping flow rate is less than the leakage flow rate, the inlet mass flow rate becomes positive and the outlet mass flow rate is negative, which indicates that the sealing capacity of the double helix seal is not enough to resist the current pressure difference. In this unbalanced state, the double spiral seal will not be able to effectively prevent fluid leakage, leakage phenomenon will occur.

Therefore, in the design of double helix seals, the balance between pumping flow and leakage flow needs to be carefully considered to ensure that the sealing device can maintain its sealing performance under various working conditions.

In the actual installation and use of double helix seal, it is found that sometimes the sealing effect is good, sometimes the sealing effect is poor or even the leakage is aggravated. It may be due to the dislocation displacement of the dynamic and static rings during assembly. The axial dislocation displacement s is the main reason for the deterioration of the sealing effect. Therefore, this part studies the influence of different axial displacements of the dynamic and static rings on the sealing performance of the double helix seal.

The inlet pressure is set to 0.1 MPa (gauge pressure), and the inner wall of the fluid is set to a rotating wall N = 10000 r/min to calculate the pressure outlet flow.

**Fig 12:**
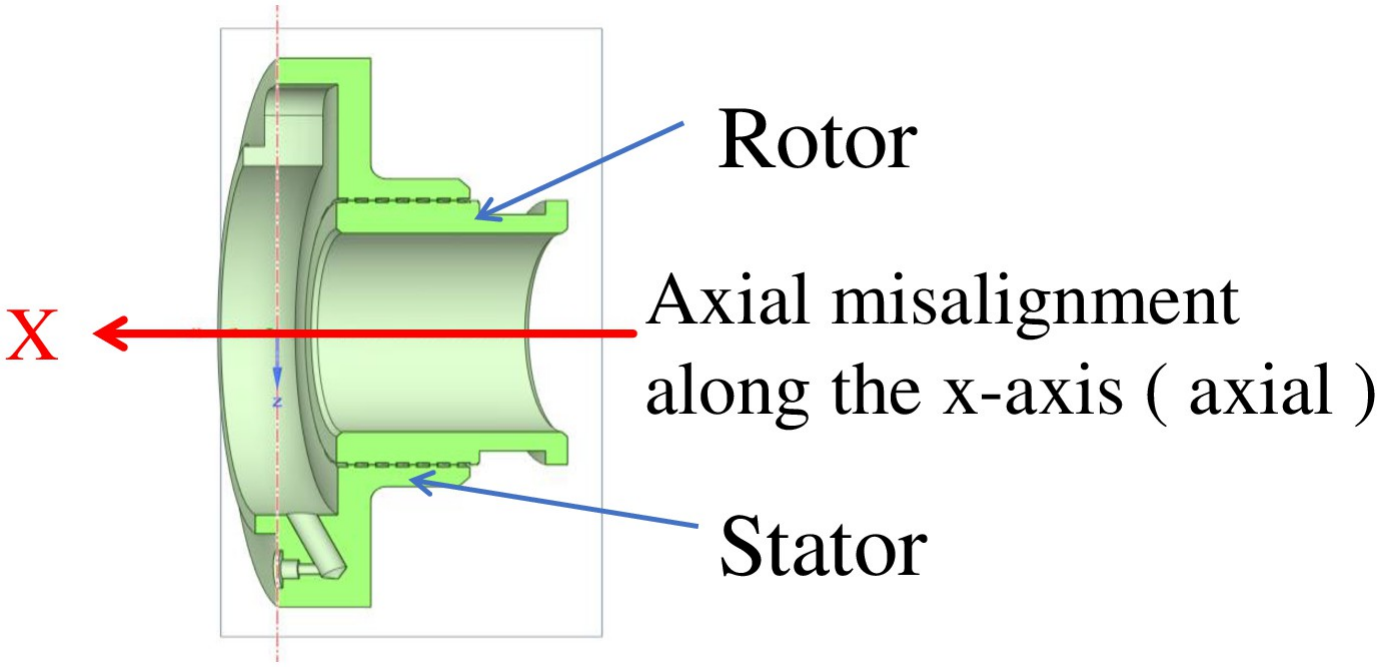
The schematic diagram of the dislocation direction of the rotor and stator.

**Fig 13:**
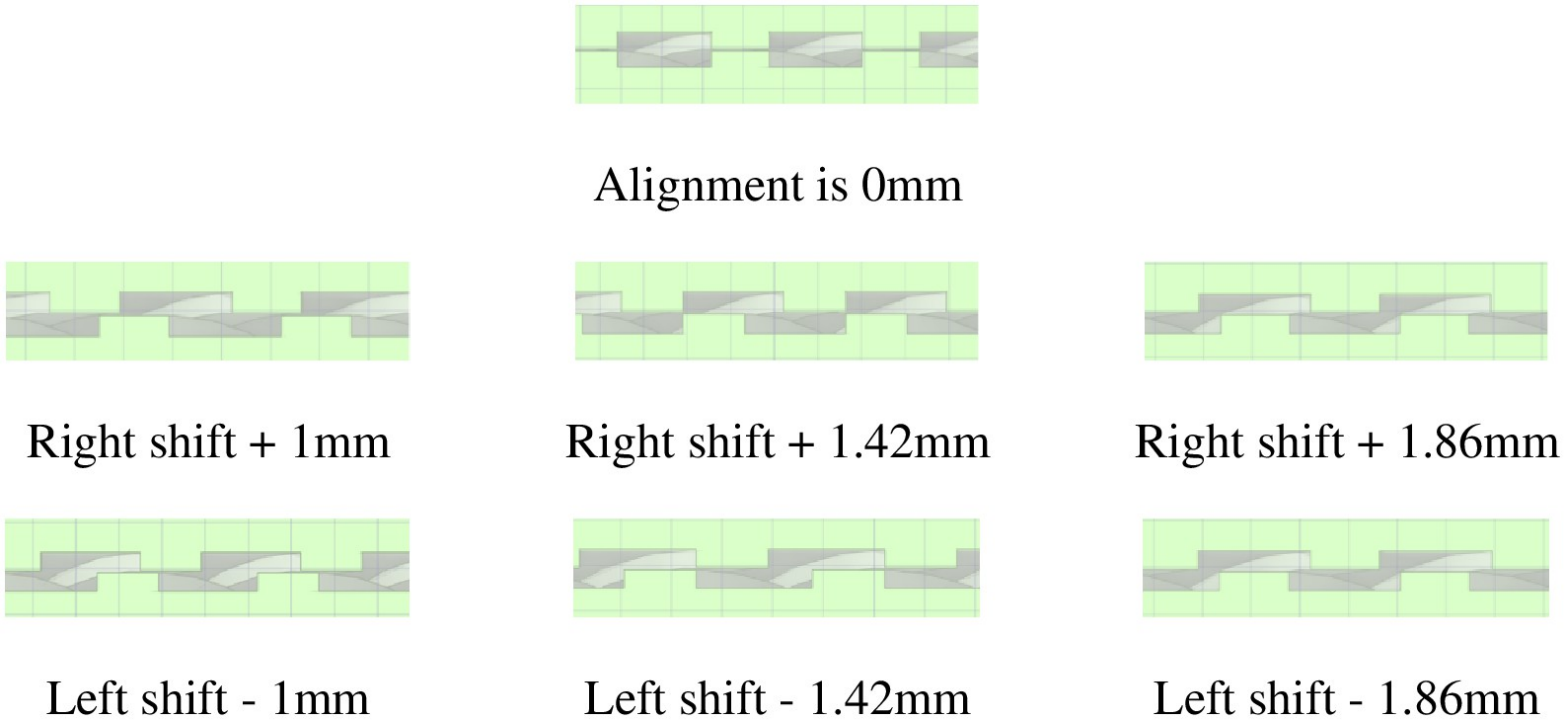
Dislocation displacement profile of the rotor and stator.

In order to more clearly show the direction and internal structure of the dislocation of the dynamic and static rings, the schematic diagram of the dislocation direction of the dynamic and static rings (Fig 12) and the dislocation displacement profile (Fig 13) are shown here.

**Fig 14:**
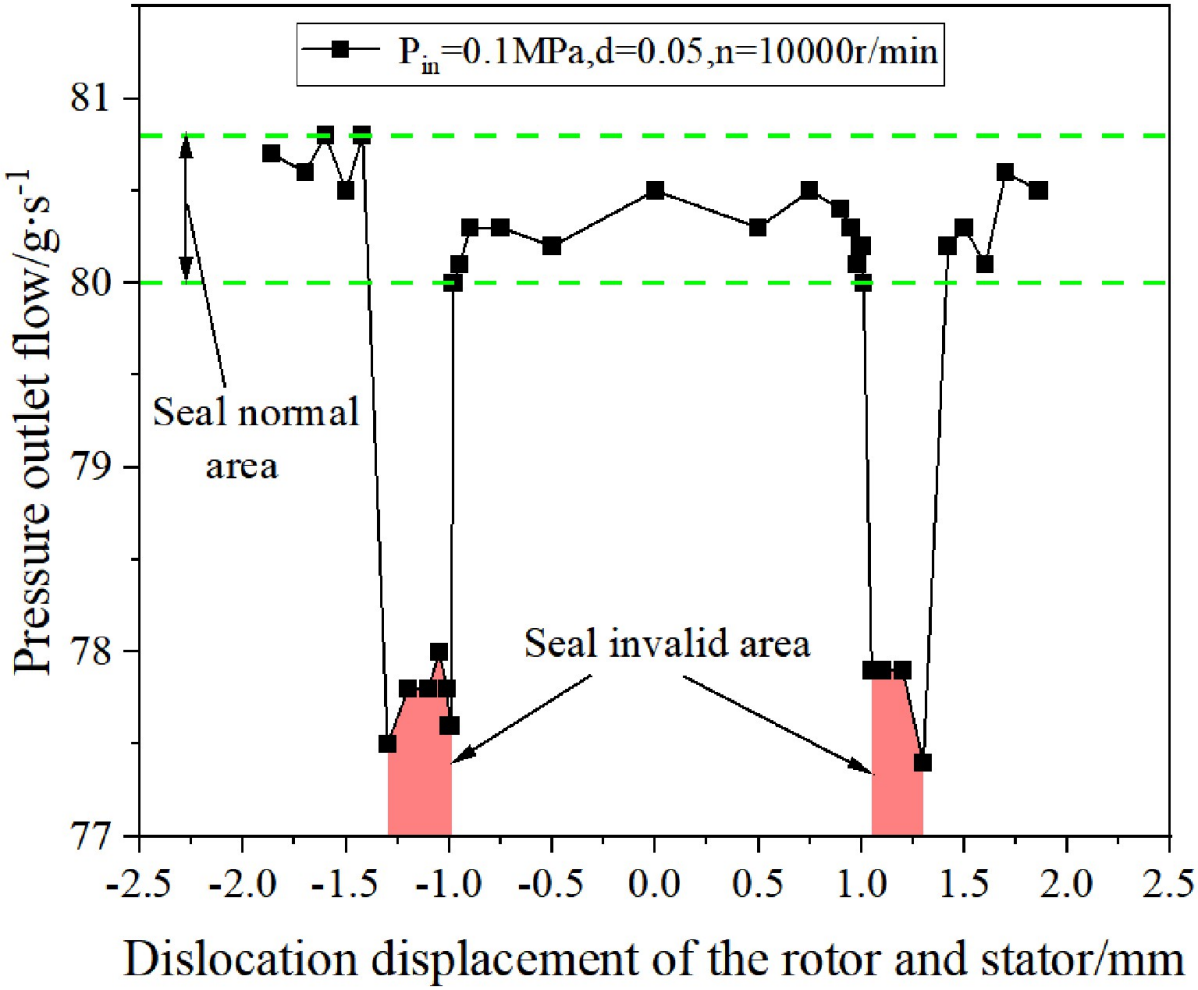
The influence of axial displacement of stator and rotor on the sealing performance of double spiral seal.

According to the calculation results, the influence of the misalignment displacement of the dynamic and static rings on the sealing ability of the spiral seal is drawn, as shown in Fig 14.

Fig 14 shows that when the displacement of the static and dynamic rings is within ± 1 mm, the sealing capacity remains basically unchanged; when the dislocation displacement of the dynamic and static rings is between ± (1 ~ 1.42) mm, the sealing capacity is reduced; when the misalignment displacement of the dynamic and static rings is between ± (1.42 ~ 1.86) mm, the sealing ability returns to the initial level. In order to facilitate the comparison of the sealing effect, this area is now divided into the sealing failure area when the dislocation displacement of the dynamic and static rings is between ± (1 ~ 1.42) mm, while other areas belong to the sealing normal area.

Under the inlet pressure of 0.1 MPa (gauge pressure), the influence of differential pressure on the sealing performance of double helix seal is inferior to the influence of pumping effect on the sealing performance of double helix seal, and the pumping effect is dominant. At this time, regardless of the axial displacement of the dynamic and static rings, the pressure outlet flow rate is always positive, which can ensure that the double helix seal plays a good sealing role.

If the inlet pressure continues to increase, the leakage flow generated by the differential pressure effect will be strengthened, and the pressure outlet flow will gradually decrease. When the inlet pressure increases to a certain value, the pressure outlet flow will change from positive to negative. At this time, the double helix seal will leak, that is, the sealing failure, which is not allowed to occur in engineering applications. Therefore, when the double helix is actually used, the axial displacement of the dynamic and static rings of the double helix seal should be reasonably controlled within a certain range. At the same time, the set inlet pressure value should not be too high to ensure that the double helix seal can give full play to the sealing effect.

### 4.3 The influence of tip clearance on the sealing performance of double helical seals

By consulting the literature, it is known that the tip clearance d is also one of the structural parameters that affect the sealing performance of the double helix seal. Now, the pressure outlet flow of the double helix seal under different tip clearances is simulated.

**Fig 15:**
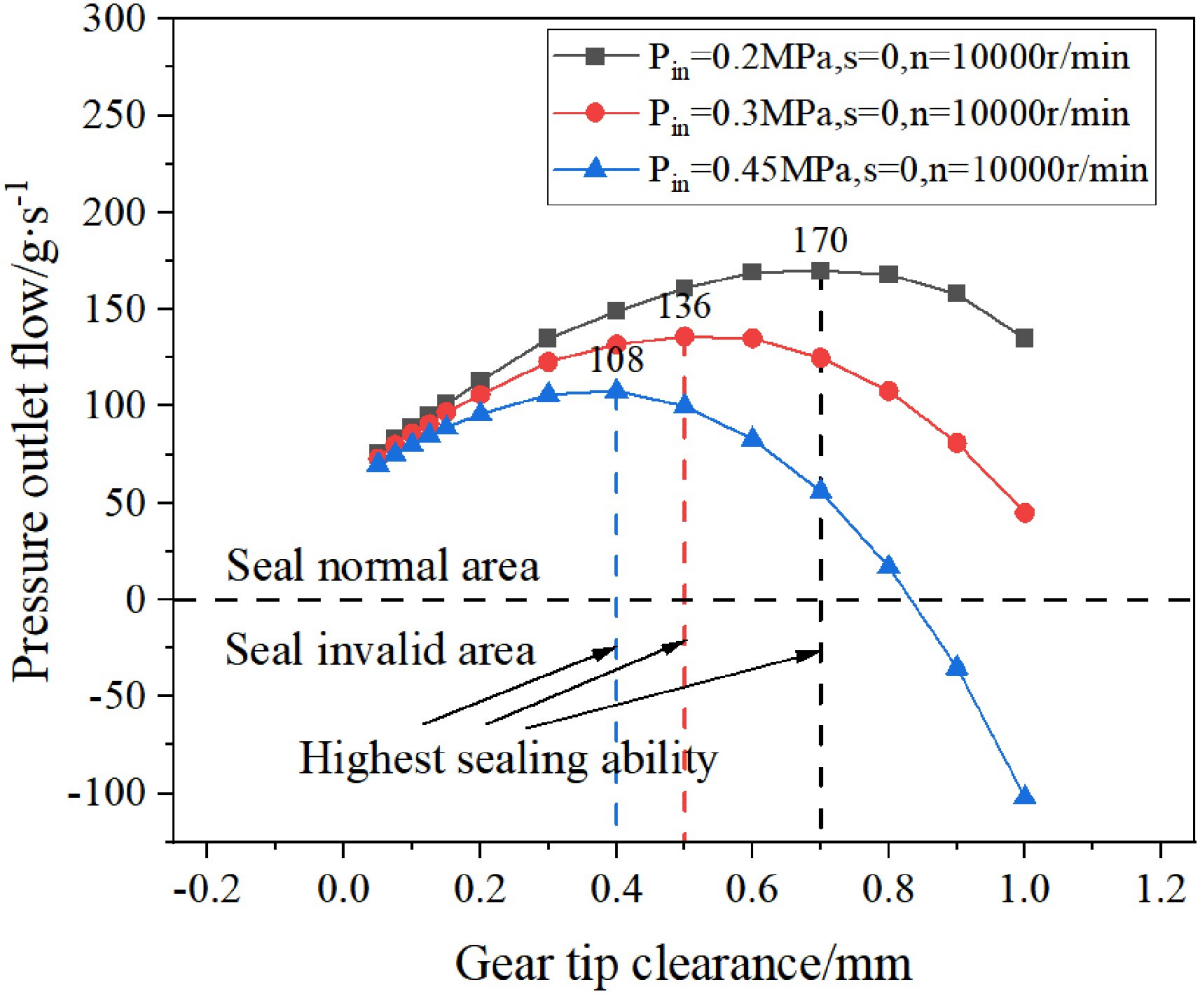
The influence of gear tip clearance on the sealing performance of double spiral seals.

According to the calculation results, the influence of the tip clearance on the sealing ability of the double helix seal is drawn, as shown in Fig 15. When the pressure outlet flow is positive, the sealing ability is good, otherwise, the sealing ability is poor.

It can be seen from Fig 15 that when the inlet pressure is constant, the sealing ability of the double-helix seal increases first and then decreases with the increase of the tip clearance, and it has the best sealing ability when the tip clearance is suitable: when P_in_ = 0.2 MPa, the tip clearance is 0.7 mm, and it has the maximum sealing ability. When P_in_ = 0.3 MPa and the tip clearance is 0.5 mm, it has the maximum sealing ability. When P_in_ = 0.45 MPa and the tip clearance is 0.4 mm, it has the maximum sealing ability.

Under the condition that the tip clearance is constant, the sealing ability of the double-helix seal decreases with the increase of the inlet pressure.

### 4.4 The influence of rotational speed on the sealing performance of double helical seals

Since the rotor speed n is one of the operating parameters that affect the sealing performance of the double-helix seal, the pressure outlet flow of the double-helix seal is simulated at different speeds.

**Fig 16:**
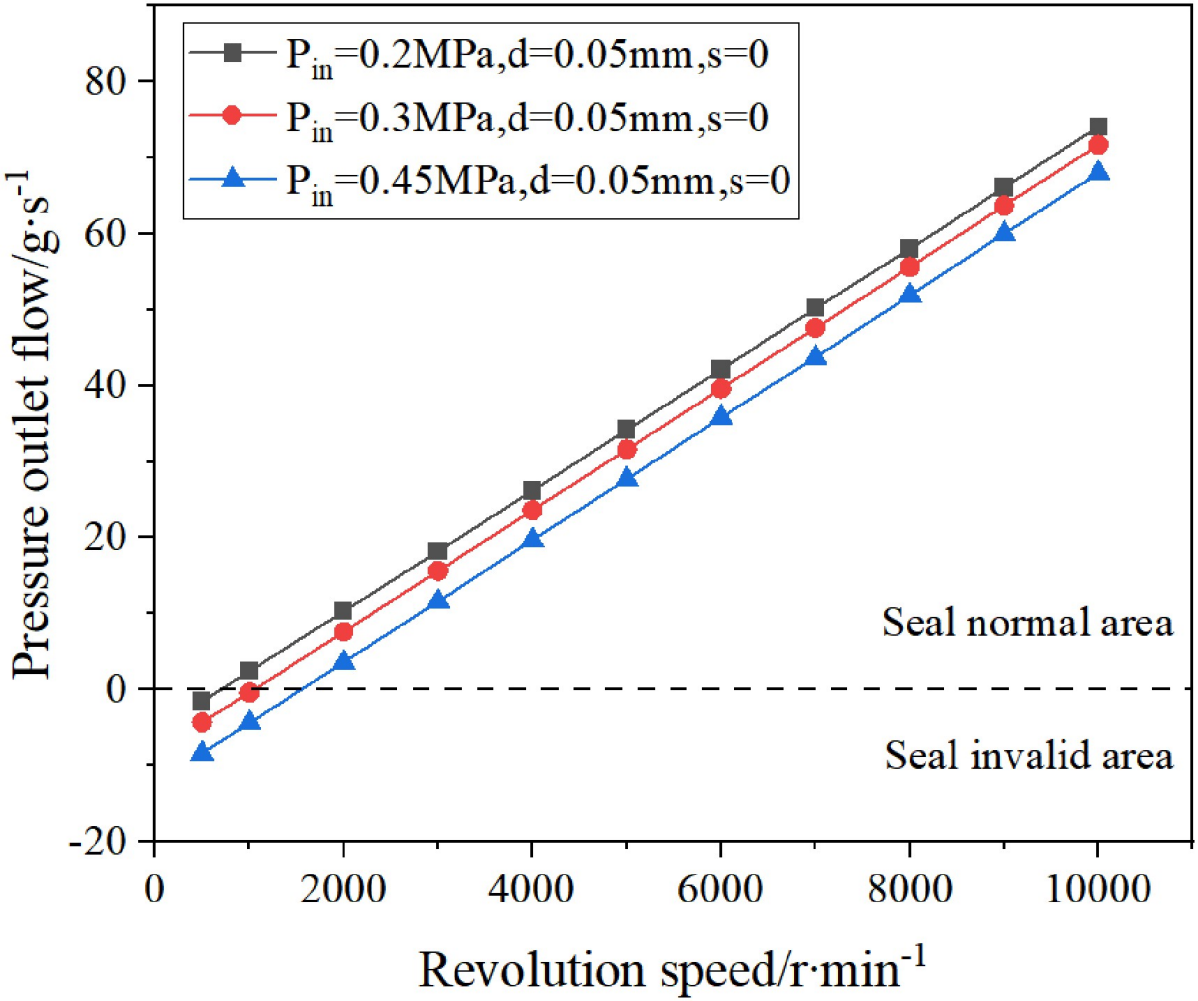
The influence of revolution speed on the sealing performance of double spiral seals.

According to the calculation results, the influence of rotor speed on the sealing ability of double spiral seal is drawn, as shown in Fig 16. When the pressure outlet flow is positive, the sealing ability is good, otherwise, the sealing ability is poor.

It can be seen from Fig 16 that under the condition of constant inlet pressure, as the rotor speed increases, the sealing capacity of the double-helix seal continues to increase; when the rotational speed is constant, the sealing capacity of the double helix seal decreases with the increase of the inlet pressure. In order to ensure the sealing ability of the double-helix seal, the speed of the rotor should be greater than a certain critical value, otherwise no pumping flow will occur and leakage will occur.

## 5. Conclusion

Based on the practical engineering problems, the geometric model of double helical seal is established. The fluid flow characteristics of double helical seal flow field are simulated and analyzed. The influence of different dynamic and static ring dislocation displacement, tip clearance and rotational speed on the sealing performance of double helical seal is calculated and analyzed. At the same time, reasonable suggestions for the design and use of double helical seal are put forward from the perspective of the influence on sealing performance and the difficulty of design and manufacture. The influence of the misalignment displacement of the dynamic and static rings, the tip clearance and the rotational speed on the sealing performance of the double-helix seal is obtained. The above research results provide a design basis for the engineering application of the double-helix seal. The innovation of this paper is that the previous research on spiral seals focuses on single spiral seals, while there are few studies on the sealing law of double spiral seals. Based on the research of single spiral seals, this paper studies the influence of axial displacement, addendum clearance and rotor speed on double spiral seals, which provides a reference for future generations to study double spiral seals. In addition, this paper also calculates and analyzes the flow field of the double helix seal, and studies the influence of the pumping effect on the seal flow field and the flow law of the fluid in the double helix seal.

Through research, the following conclusions are drawn:

1. Considering the actual installation and use of the double helix seal, the dislocation displacement of the dynamic and static rings of the double helix seal should be guaranteed between 1mm, and the sealing capacity of the double helix seal should be maintained at a high level; the inlet pressure of the double helix seal should not be set too high, otherwise it is easy to lead to seal failure and leakage.
2. Considering the sealing ability and the difficulty of design and manufacture, the tip clearance of the double helix seal should be designed between 0.5~0.8mm, and the sealing ability of the double helix seal should be maintained at a high level.
3. In order to ensure that the double helix seal can give full play to the sealing effect, the speed of the rotor should be greater than a certain critical value, otherwise there will be no pumping flow and leakage, and the speed is generally above 1000r / min. At the same time, the higher the speed is, the stronger the sealing ability is, but the speed should not be too large. The specific speed should be determined by considering the energy consumption and motor performance.

## Supporting information

S1 Data (DOCX)
