# Supplementary material for: Effect of axial misalignment and tip clearance on the performance of double spiral seals
Source: PLoS One. 2024 Dec 5;19(12):e0314912. doi: 10.1371/journal.pone.0314912 (PMC11620703; doi:10.1371/journal.pone.0314912)
Supplement: S1 Data — (DOCX) [file pone.0314912.s001.docx]

Dear Editors and Reviewers:

The original data used to construct the graph and its analysis are as follows:

Fig.1 The calculation results are compared with the literature results

Table 1 The pressure outlet flow of single spiral seal under different tip clearances

| Gear tip clearance/mm | (P_in_=0.2MPa)  Pressure outlet flow/g∙s^-1^ | (Pin=0.3MPa)  Pressure outlet flow/g∙s-1 | (Pin=0.45MPa)  Pressure outlet flow/g∙s-1 |
| --- | --- | --- | --- |
| 0.05 | -63 | -11 | 60 |
| 0.075 | -50 | 29 | 117 |
| 0.1 | -17 | 84 | 190 |
| 0.125 | 22 | 134 | 271 |
| 0.15 | 69 | 200 | 355 |

It can be seen from Fig.1 that the calculation results are basically consistent with the literature results, and the data error is within a reasonable range, which proves the correctness of the numerical method.

Fig.2 The influence of axial displacement of stator and rotor on the sealing performance of double spiral seal

Table 2 The pressure outlet flow of double spiral seal under different axial displacements

| axial displacement S/mm | Pressure outlet flow/g∙s^-1^ | axial displacement S/mm | Pressure outlet flow/g∙s-1 | axial displacement S/mm | Pressure outlet flow/g∙s-1 |
| --- | --- | --- | --- | --- | --- |
| 1.86 | 80.5 | 0.98 | 80.1 | -1 | 77.6 |
| 1.7 | 80.6 | 0.95 | 80.3 | -1.01 | 77.8 |
| 1.6 | 80.1 | 0.9 | 80.4 | -1.05 | 78 |
| 1.5 | 80.3 | 0.75 | 80.5 | -1.1 | 77.8 |
| 1.42 | 80.2 | 0.5 | 80.3 | -1.2 | 77.8 |
| 1.3 | 77.4 | 0 | 80.5 | -1.3 | 77.5 |
| 1.2 | 77.9 | -0.5 | 80.2 | -1.42 | 80.8 |
| 1.1 | 77.9 | -0.75 | 80.3 | -1.5 | 80.5 |
| 1.05 | 77.9 | -0.9 | 80.3 | -1.6 | 80.8 |
| 1.01 | 80 | -0.95 | 80.1 | -1.7 | 80.6 |
| 1 | 80.2 | -0.98 | 80 | -1.86 | 80.7 |
| 0.99 | 80.2 | -0.99 | 77.6 |  |  |

Figure 2 shows that when the displacement of the static and dynamic rings is within ± 1 mm, the sealing capacity remains basically unchanged ; when the dislocation displacement of the dynamic and static rings is between ± ( 1 ~ 1.42 ) mm, the sealing capacity is reduced ; when the misalignment displacement of the dynamic and static rings is between ± ( 1.42 ~ 1.86 ) mm, the sealing ability returns to the initial level. In order to facilitate the comparison of the sealing effect, this area is now divided into the sealing failure area when the dislocation displacement of the dynamic and static rings is between ± ( 1 ~ 1.42 ) mm, while other areas belong to the sealing normal area.

Under the inlet pressure of 0.1 MPa ( gauge pressure ), the influence of differential pressure on the sealing performance of double helix seal is inferior to the influence of pumping effect on the sealing performance of double helix seal, and the pumping effect is dominant. At this time, regardless of the axial displacement of the dynamic and static rings, the pressure outlet flow rate is always positive, which can ensure that the double helix seal plays a good sealing role.

If the inlet pressure continues to increase, the leakage flow generated by the differential pressure effect will be strengthened, and the pressure outlet flow will gradually decrease. When the inlet pressure increases to a certain value, the pressure outlet flow will change from positive to negative. At this time, the double helix seal will leak, that is, the sealing failure, which is not allowed to occur in engineering applications. Therefore, when the double helix is actually used, the axial displacement of the dynamic and static rings of the double helix seal should be reasonably controlled within a certain range. At the same time, the set inlet pressure value should not be too high to ensure that the double helix seal can give full play to the sealing effect.

Fig.3 The influence of gear tip clearance on the sealing performance of double spiral seals

Table 3 The pressure outlet flow of double spiral seal under different tip clearances

| Gear tip clearance/mm | (P_in_=0.2MPa)  Pressure outlet flow/g∙s^-1^ | (Pin=0.3MPa)  Pressure outlet flow/g∙s-1 | (Pin=0.45MPa)  Pressure outlet flow/g∙s-1 |
| --- | --- | --- | --- |
| 0.05 | 76 | 73 | 70 |
| 0.075 | 83 | 80 | 75 |
| 0.1 | 89 | 86 | 80 |
| 0.125 | 95 | 91 | 85 |
| 0.15 | 101 | 97 | 89 |
| 0.2 | 113 | 106 | 96 |
| 0.3 | 135 | 123 | 106 |
| 0.4 | 149 | 132 | 108 |
| 0.5 | 161 | 136 | 100 |
| 0.6 | 169 | 135 | 83 |
| 0.7 | 170 | 125 | 56 |
| 0.8 | 168 | 108 | 17 |
| 0.9 | 158 | 81 | -35 |
| 1 | 135 | 45 | -102 |

It can be seen from Fig.3 that when the inlet pressure is constant, the sealing ability of the double-helix seal increases first and then decreases with the increase of the tip clearance, and it has the best sealing ability when the tip clearance is suitable : when Pin = 0.2 MPa, the tip clearance is 0.7 mm, and it has the maximum sealing ability. When Pin = 0.3 MPa and the tip clearance is 0.5 mm, it has the maximum sealing ability. When Pin = 0.45 MPa and the tip clearance is 0.4 mm, it has the maximum sealing ability.

Under the condition that the tip clearance is constant, the sealing ability of the double-helix seal decreases with the increase of the inlet pressure.

Fig.4 The influence of revolution speed on the sealing performance of double spiral seals

Table 4 Pressure outlet flow of double spiral seal at different speeds

| revolution speed/  r∙min^-1^ | (P_in_=0.2MPa)  Pressure outlet flow/g∙s^-1^ | (Pin=0.3MPa)  Pressure outlet flow/g∙s-1 | (Pin=0.45MPa)  Pressure outlet flow/g∙s-1 |
| --- | --- | --- | --- |
| 500 | -1.6 | -4.3 | -8.4 |
| 1000 | 2.4 | -0.4 | -4.4 |
| 2000 | 10.3 | 7.6 | 3.6 |
| 3000 | 18.2 | 15.6 | 11.6 |
| 4000 | 26.2 | 23.6 | 19.7 |
| 5000 | 34.2 | 31.6 | 27.7 |
| 6000 | 42.1 | 39.6 | 35.8 |
| 7000 | 50.2 | 47.6 | 43.7 |
| 8000 | 58 | 55.6 | 51.9 |
| 9000 | 66.1 | 63.7 | 60 |
| 10000 | 74.1 | 71.7 | 68 |

It can be seen from Fig.4 that under the condition of constant inlet pressure, as the rotor speed increases, the sealing capacity of the double-helix seal continues to increase ; when the rotational speed is constant, the sealing capacity of the double helix seal decreases with the increase of the inlet pressure. In order to ensure the sealing ability of the double-helix seal, the speed of the rotor should be greater than a certain critical value, otherwise no pumping flow will occur and leakage will occur.
